# Development of a Hybrid Magnetic Resonance and Ultrasound Imaging System

**DOI:** 10.1155/2014/914347

**Published:** 2014-08-07

**Authors:** Victoria Sherwood, John Civale, Ian Rivens, David J. Collins, Martin O. Leach, Gail R. ter Haar

**Affiliations:** ^1^Division of Radiotherapy and Imaging, The Institute of Cancer Research and The Royal Marsden NHS Foundation Trust, 123 Old Brompton Road, London SW7 3RP, UK; ^2^Department of Clinical Magnetic Resonance, CRUK and EPSRC Cancer Imaging Centre, The Institute of Cancer Research and The Royal Marsden NHS Foundation Trust, 123 Old Brompton Road, London SW7 3RP, UK

## Abstract

A system which allows magnetic resonance (MR) and ultrasound (US) image data to be acquired simultaneously has been developed. B-mode and Doppler US were performed inside the bore of a clinical 1.5 T MRI scanner using a clinical 1–4 MHz US transducer with an 8-metre cable. Susceptibility artefacts and RF noise were introduced into MR images by the US imaging system. RF noise was minimised by using aluminium foil to shield the transducer. A study of MR and B-mode US image signal-to-noise ratio (SNR) as a function of transducer-phantom separation was performed using a gel phantom. This revealed that a 4 cm separation between the phantom surface and the transducer was sufficient to minimise the effect of the susceptibility artefact in MR images. MR-US imaging was demonstrated *in vivo* with the aid of a 2 mm VeroWhite 3D-printed spherical target placed over the thigh muscle of a rat. The target allowed single-point registration of MR and US images in the axial plane to be performed. The system was subsequently demonstrated as a tool for the targeting and visualisation of high intensity focused ultrasound exposure in the rat thigh muscle.

## 1. Introduction

In recent years there has been a move towards multimodality imaging techniques for clinical diagnosis, most commonly including various combinations of computed tomography (CT), single photon emission computed tomography (SPECT), positron emission tomography (PET), and magnetic resonance imaging (MRI). The evolution and development of these hybrid systems have been reviewed in the literature [1–5]. In many of these systems, there is an emphasis on combining anatomical and physiological information by overlaying two image data sets. Hybrid imaging reduces the reliance on image coregistration as the patient remains in the same position while both data sets are acquired. However, the systems mentioned above have disadvantages. The first is that, apart from MRI alone, they all impart some ionising radiation dose to the patient. Secondly, although tracer uptake can provide good quantitative physiological information, the spatial resolution of SPECT and PET is relatively poor compared with other modalities [5]. An additional limitation is temporal resolution. Whilst dynamic scans are possible in nuclear medicine, image noise limits the rate at which frames can be acquired. Conversely, the longer each image acquisition time, the greater the potential for patient motion.

A number of comparisons between MR and US imaging techniques for the diagnosis of disease have been made in the literature. The main limitation in many of the studies is the inability to make a true direct comparison of results, since the data had been acquired during separate studies. Lamer and Sebag [6] described the use of both MR imaging and US imaging for the diagnosis of juvenile arthritis. It is often difficult to distinguish the different arthritic conditions which can occur, but these authors concluded that MR and US imaging are the most promising techniques, although they highlighted the need for true comparative studies in this area. A study of endoscopic MR and US imaging in the staging of gastric carcinoma has been reported by Heye et al. [7]. They concluded that MR imaging is both more sensitive and more specific than US, although a lack of simultaneous imaging proved to be a limitation in comparing the data. Endoscopic US imaging was conducted preoperatively, but MR imaging was carried out on the gastrectomy specimens after resection. Postnatal imaging using MR and US has been assessed for the detection of pathology in the brain [8]. Cranial US was performed in preterm infants through the anterior fontanel at various stages following birth, in order to find and monitor disease. A single MRI scan was performed at the term equivalent age. Various conditions were seen on US, which were not detected on MRI, and vice versa. The authors highlighted the limitation of the use of a single MR examination for comparison with US imaging at different time points, since the appearance of some of the conditions on MR images changes significantly with time. A comparison between colour Doppler (CD) and MR imaging for the diagnosis of* placenta accreta* was reported by Schweel et al. [9]. *T*_1_- and *T*_2_-weighted images were acquired without contrast enhancement, and CD imaging was performed using a 7.5 MHz endovaginal transducer. The techniques showed equal sensitivity, but the accuracy and specificity of MR imaging were superior to that of CD.

The use of MR and US imaging for detecting response to therapies has also been investigated. Results of contrast-enhanced US (CEUS) and dynamic contrast-enhanced MR imaging (DCE-MRI) studies for assessing tumour vascularity following antiangiogenic therapy have been presented by Watson et al. [10]. Rats with subcutaneous tumours in the hind flank were scanned before treatment, using CEUS followed by DCE-MRI. They were then imaged 24 or 48 hours after weekly treatments. For the CEUS protocol a destruction-replenishment method was used to calculate the time for 80% replenishment of Definity contrast agent, whilst in DCE-MRI the total time to achieve 80% uptake of Magnevist gadolinium contrast agent was found. The latter method gave uptake times an order of magnitude greater than the CEUS method. Both MR and US data showed a response to treatment after 24 hours, although a direct comparison of quantitative results was not possible since the parameters measured were different. A clinical study of the response to treatment of hepatocellular carcinoma with microwave ablation has been presented by Qu et al. [11]. They compared CEUS and DCE-MRI data taken immediately after treatment and for a median follow-up period of 8 months. CEUS and DCE-MRI were equally sensitive and specific. When combined, the sensitivity increased significantly from ~86 to ~98%. Overall, combined CEUS and DCE-MRI was superior.

There are numerous publications which demonstrate applications of fused, spatially aligned multimodality imaging (image fusion). This typically involves the acquisition of MR or CT images and their transfer onto an US imaging system. Registration of images is performed manually and can be achieved using fixed points or a single fixed image plane. To monitor the position of the US imaging transducer, an electromagnetic transmitter is placed close to the imaging site, and sensors on the imaging transducer describe the changes in position during the US examination. CEUS imaging has been compared in this way with both CT and MRI as a tool for the diagnosis of renal tumours [12]. It was found that CEUS during an image fusion examination (with CT or MR data) was far more sensitive and specific in detecting tumours than MR, CT, or US imaging alone. Rennert et al. [13] used CEUS coupled with either contrast-enhanced CT (CECT) or DCE-MRI to study liver tumours. Fusion imaging allowed more specific diagnoses in 15 of 21 patients and resulted in a change in therapeutic strategy for 12 of 84 patients. There are a number of other applications for which this technique has shown promise, such as in prenatal imaging [14] and biopsy guidance for musculoskeletal tumour diagnosis [15] and in the characterisation of tumour vascularity [16].

The main disadvantage of both fusion imaging and comparative MR and US studies in general is the necessity for multiple examinations. There can be no inherent form of positional registration between the image data sets. Through careful planning and positioning, simultaneous MR-US imaging could lead to improved and more efficient diagnosis of disease, increasing the breadth of diagnostic imaging. It could also allow comparative studies to be conducted more reliably, since the anatomical and physiological features and processes become identical for both image types. The limitation would be a practical one, as it would require sufficient space within the magnet bore to move the US transducer, and the ability to perform freehand scanning would be limited, unless performed immediately prior to the MR scan.

A number of systems are already in place for conducting simultaneous MR and US imaging. Studies in the literature range from proof of principle [17] and motion correction of MR data using ultrasound imaging [18–20] to interventional procedures such as breast biopsy guidance [21]. Furthermore, therapeutic applications have been explored. A study of artefacts in MR thermometry during RF ablation has been conducted [22]. US images were used to visualise thermally induced cavitation bubbles close to RF applicators embedded in gelatin phantoms and* ex vivo* tissues. A model based approach was then used to correct for inaccuracies in MR thermometry caused by the susceptibility artefacts introduced in phase images by the cavitation bubbles. Arvanitis et al. [23] describe a study in which an ultrasound imaging array is used passively to detect acoustic signals originating from acoustic cavitation bubbles during MRgHIFU exposure in the brains of large mammals. Pulsed HIFU exposures were used to disrupt the blood brain barrier (BBB) in the presence of US contrast agents. Maps of cavitation activity were produced by reconstructing signals received by 64 elements of a linear array transducer placed at right angles to the direction of the HIFU beam. A study by Petrusca et al. [24] has demonstrated simultaneous MR and US imaging using modified clinical imaging apparatus, and the authors have explored some of the interactions taking place when performing B-mode US alongside 1.5 and 3 T MR imaging, for the purposes of motion monitoring. At 3 T they have shown a 3–6% SNR penalty in MR images through the acquisition of US B-mode data simultaneously, compared with that seen when the US transducer was present, but not actively imaging. Since the majority of MRI systems currently used routinely in clinical diagnosis are 1.5 T systems, it would be appropriate to evaluate the same effects on clinical MRI sequences at 1.5 T, where the inherent SNR is significantly lower. Furthermore, the impact due to the presence of the US transducer within the MR scanner has not been explored.

One of the primary advantages of US imaging is its real-time capability. For this reason, the majority of studies involving simultaneous MR and US imaging for HIFU monitoring applications have used the US data for the purposes of motion compensation [25, 26]. Another strength of US imaging systems is their ability to provide accurate real-time measurements of blood flow. Doppler US has not yet been exploited in a hybrid imaging situation. The use of Doppler US during MR-guided HIFU would be advantageous from a treatment planning perspective, where perfusion and large vessels may disrupt the delivery of heat to tissues, and in applications involving vascular occlusion. As Doppler US is very sensitive to motion, an exploration of the effects of simultaneous MR imaging on the quality of Doppler data is required.

This paper describes the development of a system for performing simultaneous MR and US imaging, with a view to improving guidance and monitoring of thermal ablation using HIFU. Combining B-mode, Doppler US and MR image data would provide information that might have the potential to increase the efficiency of treatment regimes by indicating the presence of cavitation bubbles and/or the presence of flow in nearby blood vessels. This study is the first to explore in detail the interactions between 1.5 T clinical MR imaging and different modes of US imaging, including Doppler. SNR and image distortion effects are studied as a function of distance to provide information on the appropriate arrangement of equipment for hybrid imaging studies of this kind. The* in vivo* use of the system is demonstrated during HIFU exposure of muscle tissue in the rat thigh. This paper adds to the breadth of previous literature on hybrid MR-US imaging for the monitoring of therapeutic applications, which, in the most part, have not demonstrated the use of US data beyond motion compensation.

## 2. Methods

### 2.1. Imaging Equipment

The MR scanner used in this study was a 1.5 T Siemens Avanto clinical scanner used routinely for diagnostic examinations. Due to the arrangement of equipment required for HIFU studies, the head array was used as the receive coil for all experiments. A Siemens Antares clinical US scanner was used to provide the US imaging within the MR scanner bore. Since the US scanner cart could not be taken into the magnet room, the manufacturer provided a customised curvilinear 1–4 MHz US imaging transducer with an 8-metre long cable (CH4-1), thus allowing the cart to be located outside the MR room. For the purposes of demonstrating the feasibility of hybrid MR-US imaging for future clinical use, it was considered important to use clinically applicable imaging equipment, and this informed the choice of scanners. The specific choice of US imaging probe was made by the manufacturer, who modified only the lowest frequency transducer, to minimise the effects of attenuation due to the additional cable length. The attenuation increases with increasing frequency, and therefore a 1–4 MHz abdominal probe was the least likely to suffer prohibitive levels of image degradation.

### 2.2. Investigation of Noise during Simultaneous Data Acquisition

**Figure 1:**
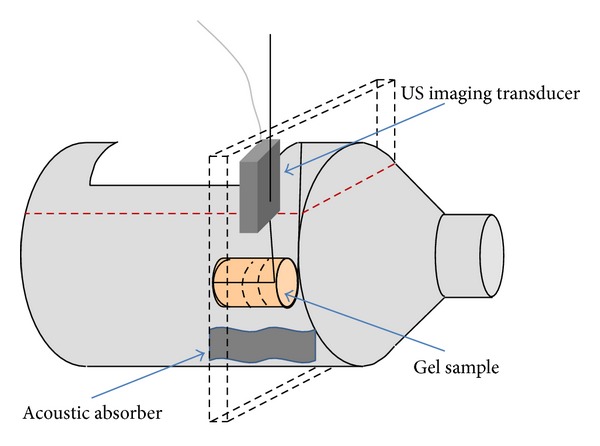
Bottle containing filtered, degassed water, gel sample, and US imaging transducer. The plane of the coincident MR and US images is marked with black dashed lines, which also indicate the position of this plane in the gel sample. The approximate water level is shown by a red dotted line.

**Figure 2:**
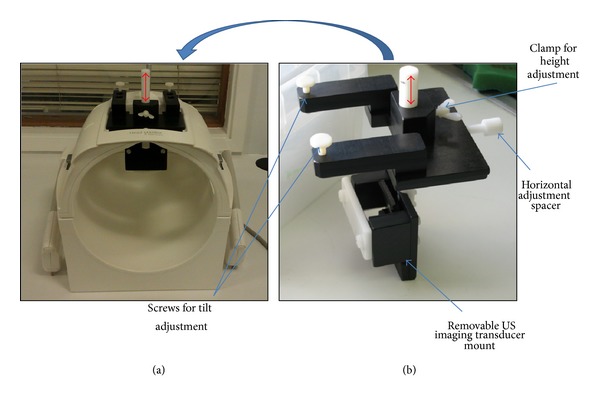
(a) Siemens Avanto head coil with US imaging transducer mount, and (b) a side view of the US imaging transducer mount, connected by an adjustable vertical rod (red double headed arrows).

Two initial experiments to assess the influence of each imaging modality on the other during simultaneous data acquisition were performed. A plastic bottle with a slot cut in its side was used as a water tank, as shown in Figure 1. It was filled with 5 *μ*m-filtered tap water, which had been degassed by placing under vacuum at <−635 mm Hg for at least 12 hours prior to each experiment. A Perspex sample holder was attached to one side of the opening in the bottle, so that a cylindrical sample of PVA cryogel, based on a recipe developed by Fromageau et al. [27] (10% PVA and 5% cellulose by weight, with 0.4 g/L Gd-DTPA, 3 freeze-thaw cycles, 4.5 cm diameter, and 4.5 cm length), could be held under water. By placing the bottle neck-first into the MR head coil, it was possible to acquire axial image slices through the gel, as shown by the dashed slab surrounding the bottle in Figure 1. The US transducer was mounted in the arrangement shown in Figure 1 using a dedicated holder which was inserted, via a vertical rod, into a mounting platform which had been built specifically to sit on the head coil, as shown in Figure 2. This allowed the US imaging transducer to sit over the gap in the top of the head coil, facing down into the water. Its orientation allowed imaging of a slice through the gel in the same orientation as the axial MR slice described previously. Once a cross-sectional image of the sample had been visualised on B-mode, the MR scanner positioning lasers were used to place the US transducer at the isocentre of the magnet. Since the mounting platform had been machined to hold the transducer in a vertical position, it was assumed for the purposes of this part of the study that the MR and US imaging planes were coincident. For the* in vivo* work, additional steps were necessary to verify the alignment, and these are described along with the associated methods.

The first experiment investigated the potential influence of RF noise from the US scanner on MR images during hybrid MR-US imaging, with and without implementing a simple shielding technique. The two MR sequences used most frequently for clinical diagnostic imaging in our MR department were chosen for this study. The first was a *T*_1_-weighted fast low angle shot (FLASH) sequence (TR/TE 221/7.15 ms, flip angle 70°, slice thickness 6 mm, field of view 150 × 200 mm, matrix 192 × 256 pixels, and bandwidth (BW) 230 Hz/pixel) and the second was a turbo spin echo (TSE) *T*_2_-weighted sequence (TR/TE 4000/102 ms, flip angle 150°, slice thickness 6 mm, field of view 150 × 200 mm, matrix 384 × 512 pixels, echo train length 29, and BW 260 Hz/pixel). MR data were acquired using these 2 sequences, with the US transducer connected to the US scanner, which was switched on but had the imaging frozen. The US transducer face was 7 cm above the top of the gel sample. This was then repeated whilst simultaneously acquiring B-mode, colour Doppler (CD), power Doppler (PD), and spectral Doppler (SD) US data. Finally, the same set of imaging protocols were repeated once the entire transducer head and cable had been wrapped in 10 *μ*m thick aluminium catering foil (WBS, Amersham, Bucks, UK), with a layer of US transmission gel (Aquasonic 100, Parker Laboratories Inc., New Jersey, USA) coupling the transducer face to the foil. At the door of the MR scanner room, the foil was placed in contact with the RF cage by partially closing the door such that the catch engaged but the cable was not compressed. Settings for the various US imaging modes are summarised in Table 1. In all experiments the output power was fixed at 100% and the time gain compensation (TGC) settings were adjusted to give uniform image brightness through the phantom material. Finally, MR images were acquired in the absence of the US transducer and with the scanner room door closed.

The impact of US imaging on MR data was quantified using the signal-to-noise ratio (SNR) calculated in a circular region of interest (ROI) located wholly within the cryogel. SNR was calculated using the mean pixel value within the ROI from a single slice (the signal, *S*) and the standard deviation of pixel values from the same ROI in a subtraction image of two identical slices taken one after the other (the noise, *N*). These two parameters were combined using (1) [28]. The associated uncertainty was calculated from the percentage uncertainty in the ROI in a single image frame:

(1) SNR=2SN.

With the aluminium foil present, the impact of acquiring MR data during B-mode imaging was investigated by calculating the B-mode SNR from images of the gel obtained before and during MR imaging. Prior to MR scanning, a clip of 75 B-mode frames was acquired. This is the default size for a clip. This was then repeated during both MR pulse sequences. After exporting the data from the scanner in DICOM format, SNR values were calculated using frame 1 and each of frames 2–75, resulting in 74 different SNR measurements for each of the 3 data sets. Clips of the same size were also acquired for CD, PD, and SD modes. The impact of MR data acquisition on CD and PD images, and on SD data, was assessed qualitatively by visually inspecting those frames from each mode which contained the largest artefacts, since the noise from the MR scanner was time varying. Using the B-mode data acquired whilst the MR scanner was idle, the impact of the single layer of aluminium foil over the transducer face was quantified by comparing B-mode SNR in the gel for the two experiments performed with and without the foil. For all subsequent experiments the US imaging transducer was wrapped in aluminium foil.

### 2.3. Magnetic Susceptibility Effects of the US Transducer

The aim of the experiment was to determine the distance required between the US imaging transducer and the gel sample in order to avoid the effects of magnetic susceptibility artefacts produced by the transducer in different types of MR image. Using the height adjustment on the US transducer mount, the distance between the top surface of the cryogel sample and the front face of the transducer was varied between 0 and 7 cm, in 1 cm increments. This range allowed evaluation of the potential to image a sample in direct contact with the transducer face and at the greatest separation that the available space inside the magnet would allow. Throughout this experiment the US transducer was connected to the US scanner, which was switched on, but the imaging was frozen. Axial MR images were acquired through the centre of the gel using the 2 sequences described previously. In addition, 2 further echo planar imaging (EPI) based sequences were investigated: a gradient echo (GRE) segmented EPI (seg-EPI) sequence used for MR thermometry (TR/TE 70/15 ms, flip angle 60°, slice thickness 5 mm, field of view 180 × 180 mm, matrix 128 × 128 pixels, and BW 601 Hz/pixel) and a diffusion-weighted imaging sequence (TR/TE 5000/123 ms, flip angle 180°, slice thickness 6 mm, field of view 208 × 208 mm, matrix 128 × 128 pixels, BW 1953 Hz/pixel, and b-values 0 & 800) from which ADC maps were calculated using a single exponential model. These types of sequences are known to be sensitive to susceptibility induced signal loss and distortions [29, 30] and are relevant to future HIFU studies, so they were used to test this potential limitation of the hybrid imaging system.

Finally B-mode US images were acquired at each transducer position whilst the MR scanner remained idle, to determine the change in B-mode SNR with distance from the sample. The B-mode frequency and image depth were fixed at 4 MHz and 16 cm, respectively, and the gain was set to 0 dB. The maximum frequency (4 MHz) was chosen in order that the spatial resolution would be maximised in future experiments. A total depth of 16 cm was chosen as it covered the entire depth of the tank. A single focal position was used, and its depth was varied with transducer-sample separation to achieve the best lateral resolution at the centre of the sample.

Spatial distortion was quantified in the MR images by measuring the deviation of the cross-section of the gel sample from a circle. Since the samples were moulded using rigid cylindrical containers, their cross-section should have been very close to circular. An ellipse was placed over each image slice in turn, and its size and position adjusted to best fit as much of the outline of the cryogel as possible. Its dimensions were then recorded and (2) was used to calculate the eccentricity, where *a* and *b* are the long and short axes of the ellipse, respectively. This procedure was used to overcome the limitations of measuring the outer edges of the gel in images where they are obscured partially by the susceptibility artefacts:

(2) $$\begin{array}{c} \varepsilon =\sqrt{1-{\left(\frac{b}{a}\right)}^{2}}. \end{array}$$

For a circle *ε* = 0, and as a shape becomes more elliptical *ε* tends towards 1. This manual assessment process was repeated 3 times for each image, to allow calculation of uncertainty using the standard deviation in the results.

### 2.4. Hybrid MR-US Imaging of HIFU Exposure* In Vivo*

#### 2.4.1. Preliminary Alignment

**Figure 3:**
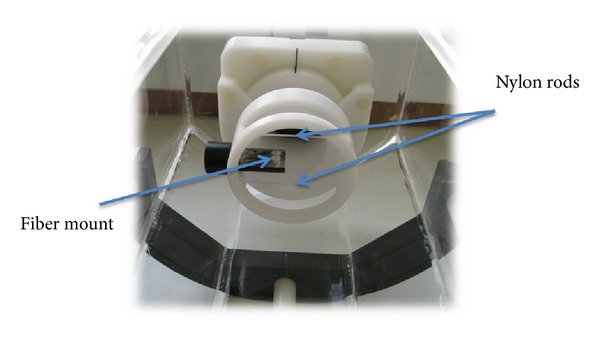
Perspex tank with integrated phantom mount and alignment phantom used to permit inherent registration of MR and US imaging planes.

Since the system was to be used for HIFU treatment visualisation, it was necessary to ensure the imaging planes were accurately aligned, without angulation of the US imaging transducer. Furthermore, it was necessary to align the imaging planes with the focal plane of the HIFU beam. To this end, a Delrin alignment phantom, containing 2 nylon rods and a mount to hold a fibre-optic hydrophone sensor, was constructed. This is shown in Figure 3. Acoustic windows at the top and bottom allowed the US imaging beam to pass through the phantom. A Perspex tank with integrated mounting block had been constructed for the purpose of mounting first the phantom and, subsequently, an anaesthetised rat.

Prior to* in vivo* imaging, the phantom was imaged using B-mode US and *T*_2_-weighted MRI, using the imaging parameters described above. After aligning the MR scanner lasers with the nylon rods, the US transducer mount position and alignment screws were adjusted to give the brightest image of both rods, ensuring the plane was aligned horizontally and vertically relative to the tank. The rods were situated at 4 and 8 cm depths in the B-mode image. *T*_2_-weighted MR image slices with 1 mm thickness were then acquired through the phantom, to determine the correct slice position which coincided with the US image plane. The position of the US transducer mount's horizontal adjustment spacer was fixed, to allow repeatable repositioning of the imaging transducer, since it was necessary to remove and replace it whilst positioning the subject.

HIFU exposures of 5 s duration at 1.7 MHz were provided by a 1.7 MHz single element MR-compatible transducer (H148MR, 64 mm diameter with 20 mm central aperture, 63 mm focal length, Sonic Concepts, Washington, USA) with 6 dB focal peak dimensions of 9.9 × 1.2 mm. It was mounted on an MR compatible micrometer gantry, built in-house. A timer box, also built in-house, was situated in the MR scanner control room and used to send a 5 V transistor-transistor logic (TTL) trigger signal to an arbitrary waveform generator (HP33120A, Agilent, USA), via a BNC cable which had been fed through the roof space into the scanner's plant room. Signals were amplified (55 dB, ENI A300, E&I, Rochester, USA) and sent into the magnet room via a voltage and current pick-off box and a 63 MHz high power notch filter (both built in-house) connected to a BNC feed-through panel in the RF cage. Within the magnet room, signals were sent through a BNC cable into the HIFU transducer via its associated impedance matching network. A fibre-optic hydrophone system (Precision Acoustics, Dorchester, UK) was situated in the MR control room, controlled by a laptop. A single 10-metre optical fibre hydrophone was fed from this into the scanner room through a waveguide and positioned in the alignment phantom. HIFU bursts (40 cycles, 1.38 ± 0.08 MPa, pulse repetition frequency 100 Hz) detected by the hydrophone were visualised on an oscilloscope (Waverunner 64Xi, 600 MHz, 10 GS s^−1^, Teledyne Lecroy, Berkshire, UK), and the micrometer gantry was used to adjust the HIFU transducer position by hand, until the signal amplitude was maximised.

#### 2.4.2. *In Vivo* Experimental Methods

**Figure 4:**
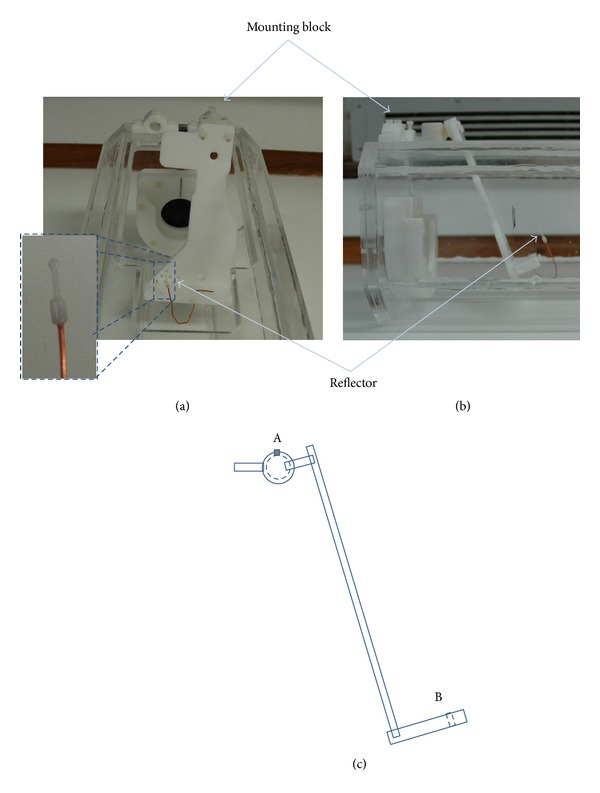
(a) Front and (b) side views of the Perspex tank with Delrin mounting blocks holding the rat platform. The reflector used for image and HIFU alignment is shown on the left (insert). (c) Schematic diagram depicting the side view of the rat platform. “A” indicates the universal joint to allow flexible positioning of the animal, with screw to secure it in position. “B” indicates a hole in the base plate (dashed lines), through which a cable tie was threaded in order to hold the right foot in position, preventing the animal from moving and therefore maintaining imaging access.

Imaging and HIFU were performed* in vivo* in accordance with Home Office licences. A female Sprague Dawley rat was anaesthetised using 60 mL/kg pentobarbitone, giving 2-3 hours of anaesthesia. After inner thigh hair removal, the subject was mounted onto a custom built holder (Figure 4) with its foot pulled out and secured gently using a small cable tie, threaded through a hole in the base of the mount (labelled B in Figure 4(c)). PD US flow measurements in the femoral artery were acquired outside the magnet room using both the MR compatible CH4-1 probe and an additional P10-4 probe, which provided better spatial resolution. A 2 mm diameter sphere was 3D printed in VeroWhite (Objet, Germany) and attached to one end of a piece of copper wire (also 2 mm diameter), the other end of which was threaded through another hole in the mount's base. Using the flexible wire, the reflector was placed over a target position on the front surface of the thigh, over the muscle, but away from the bone.

Once the US transducer mount and alignment phantom had been removed, the mount was attached to the Perspex tank at point A (Figure 4(c)), and the US imaging mount was replaced. *T*_1_- and *T*_2_-weighted transverse MR images were taken through the thigh of the animal and the VeroWhite target. *T*_2_-weighted images allowed the target to be clearly seen as a black spot in contrast with the surrounding water. *T*_1_-weighted images were then used to assess the position of the target relative to anatomy such as bone, muscle, and blood vessels. The slice position coinciding with the VeroWhite target was noted, and a translation of the US imaging transducer in the *z* direction was performed, to find the position of the target on B-mode. An US image frame was captured at this position. Subsequently the target was pulled away from the thigh, and both imaging planes and the HIFU transducer position were adjusted to target the thigh muscle 5 mm beneath the skin surface.

Images were acquired using B-mode US and all four MR image sequences described previously, both prior to and immediately following a 5 second continuous HIFU exposure at a focal peak intensity of 1330 W cm^−2^. MR thermometry was performed using the segmented EPI sequence, producing a map of temperature rise every 0.8 s throughout the exposure. Two pre-HIFU frames and 30 post-HIFU frames were also acquired.

RF US data were transferred from the scanner to a PC for postprocessing. These data underwent a scan conversion using Matlab code provided by Siemens. This process converted a square matrix of voxel intensities into a sector, allowing it to be displayed as it appeared on the scanner display. Pre-HIFU baseline B-mode images were subtracted from those taken immediately after HIFU to generate maps of echogenicity change, indicative of bubble formation in the tissue. MR images were transferred to the same workstation, and EPI phase images were processed for calculation of temperature rise maps using a Matlab program written in house.

Although a number of sophisticated methods have been developed previously for performing MR and US image registration [31, 32], it was considered appropriate to use a single-point registration to combine the MR and US image data, due to the inherent spatial registration achieved through the alignment process. Using a further in-house Matlab program, the VeroWhite target position was identified in both B-mode US and *T*_2_-weighted MR images using a mouse click, and from these it was possible to calculate the image translation required to superimpose one over the other. Further translations, taking into account the in-plane pixel dimensions, were calculated to allow both US images and MR temperature maps to be displayed over *T*_1_-weighted images.

## 3. Results

### 3.1. MR and US Image Noise

**Figure 5:**
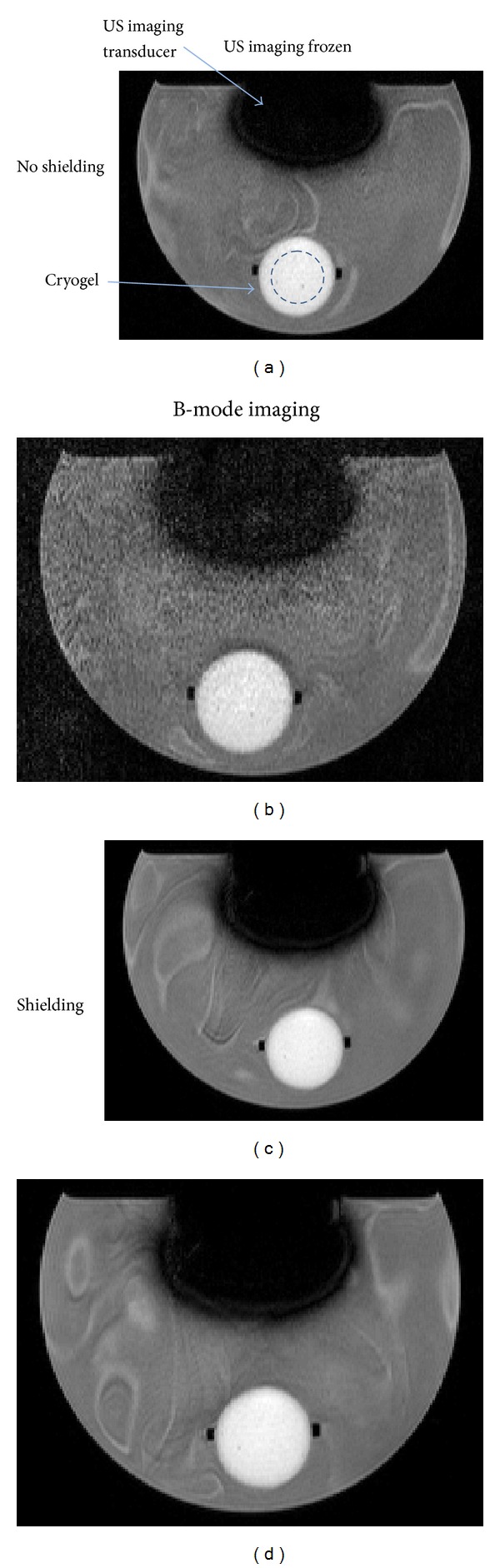
*T*_1_-weighted images of a PVA cryogel sample mounted in the water tank, with the US imaging transducer mounted above it. The phase encoding direction was from top to bottom in these images, and parallel imaging was used, with signals combined using adaptive weighting. Images were taken without (a and b) and with (c and d) aluminium foil shielding of the US probe and cable, whilst the US scanner was frozen (a and c), and during B-mode imaging (b and d). The blue circle marked within the cryogel in (a) represents the circular ROI used for SNR calculations. Each image shows a field of view (FOV) measuring 22 × 16 cm.

**Figure 6:**
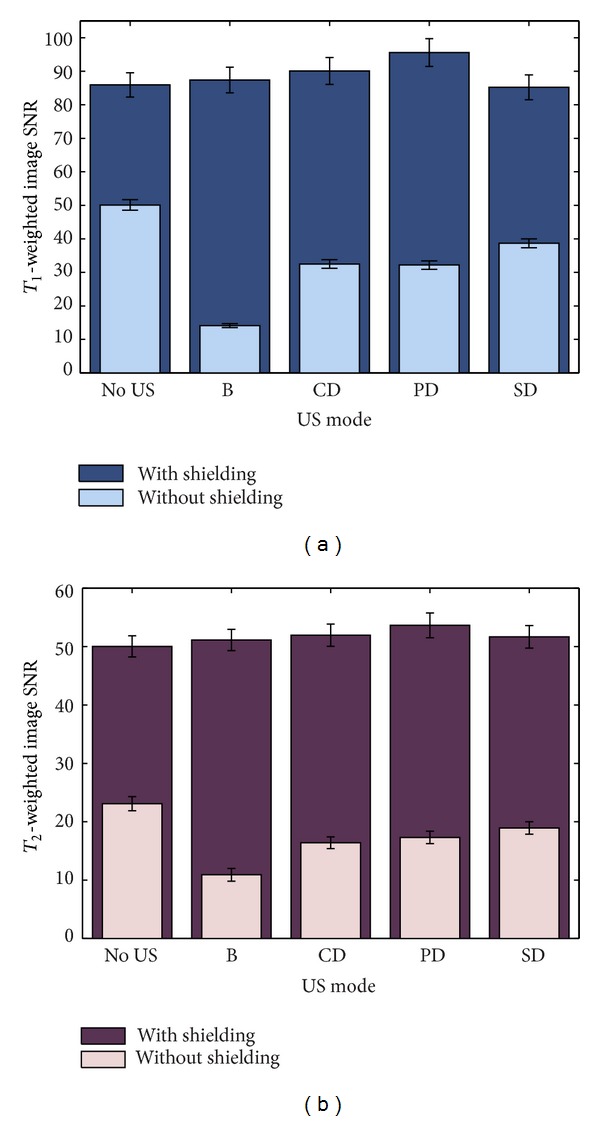
SNR in a ROI in cryogel for (a) *T*_1_-weighted (FLASH) and (b) *T*_2_-weighted (TSE) MR images obtained during various modes of US image acquisition, showing values with and without aluminium foil shielding of the US probe and cable. Error bars represent the percentage uncertainty in intensity within the ROI in a single image frame in each case.

Figure 5 illustrates the appearance of noise from US imaging in MR images, showing *T*_1_-weighted axial image slices through the US imaging transducer and the cryogel sample, without (a-b) and with (c-d) aluminium shielding on the probe and cable. These images were acquired both prior to (a and c) and during (b and d) B-mode US imaging. The lack of a clearly defined outline of the US transducer face indicates surrounding signal loss due to a susceptibility artefact. Using the dimensions of the imaging transducer head (75 mm wide at the widest point), it can be seen that the artefact extends approximately 1 cm from its outer edge. SNR values for the ROI marked in blue are plotted in Figure 6 for the various combinations of MR and US imaging sequences/modes, with and without aluminium foil shielding of the US probe and cable.

In the complete absence of the US imaging transducer, the SNR within the gel in the *T*_1_-weighted images was 95.0 ± 3.6 and in the *T*_2_-weighted images was 106.1 ± 2.7. For the *T*_1_-weighted images, this was not significantly different from the average SNR measured with the US transducer present and shielded with aluminium foil, suggesting that the passive influence of magnetic susceptibility is minimal at this distance. In contrast, for the *T*_2_-weighted images, the SNR values measured with the US transducer present and shielded were on average 50% lower than those in the complete absence of the transducer.

**Figure 7:**
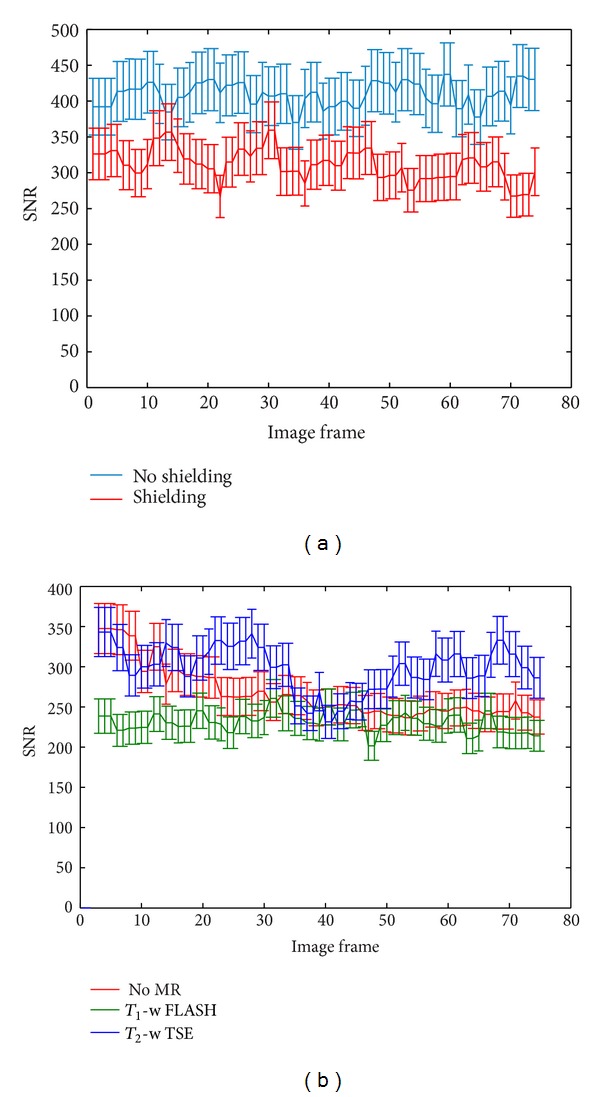
(a) B-mode SNR for all frames of clips taken with and without aluminium foil shielding on the transducer and cable, whilst the MR scanner was idle. (b) SNR of the gel sample in B-mode images taken whilst the MR scanner was idle and whilst FLASH and TSE sequences were in use. A total of 74 measurements were made for each data set.

US probe shielding resulted in an increase in SNR of a minimum of 72% (FLASH with no US imaging) and a maximum of 520% (FLASH with B-mode imaging).  Figure 7(a) indicates that the associated B-mode SNR was reduced when shielding was used. This was calculated to be a 24% reduction on average, and the two groups of pixel values, with and without shielding, were found to be highly significantly different (*P* < 0.001) using a Student's *t*-test.

**Figure 8:**
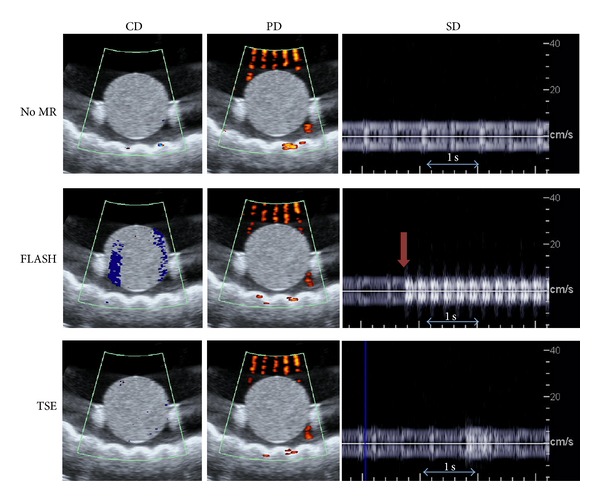
CD and PD images and SD traces, taken before and during *T*_1_- (FLASH) and *T*_2_-weighted (TSE) imaging. The CD and PD images shown were those worst affected by artefacts out of a set of 75 frames. The vertical arrow (RHS, middle image) indicates the time point at which the FLASH sequence began. The horizontal arrows in the SD images indicate a 1-second time period. The horizontal echogenic structure visible beneath the gel is an acoustic absorber, placed in the tank to minimise reflections during US imaging. The extent of the B-mode FOV shown in these images is 9 × 9 cm.

B-mode SNR data are shown in Figure 7(b) for 74 frames in each of 3 data sets, taken whilst the MR scanner was idle and repeated during the FLASH and TSE sequences. SNR values from the B-mode images are approaching an order of magnitude greater than those in the various MR images. At no point do the values during MR acquisition fall below the lowest values taken whilst the MR scanner was idle.  Figure 8 shows CD and PD images and samples of SD traces, taken with the MR scanner being idle and during both FLASH and TSE MR imaging sequences.

Colour artefacts appear in the CD image during the FLASH sequence, equivalent to approximately 5 cm/s, and in all PD images, although in the PD images the artefacts do not propagate into the gel sample. More detail is seen in the SD data, where the onset of the FLASH sequence is clearly visible against the inherent noise from the idle magnet (vertical arrow), after which a quasi-continuous level of noise is detected. A single burst of noise is visible in the SD data during the TSE sequence, and these were observed to occur every 4 seconds—equivalent to the TR.

### 3.2. Transducer-Sample Separation

**Figure 9:**
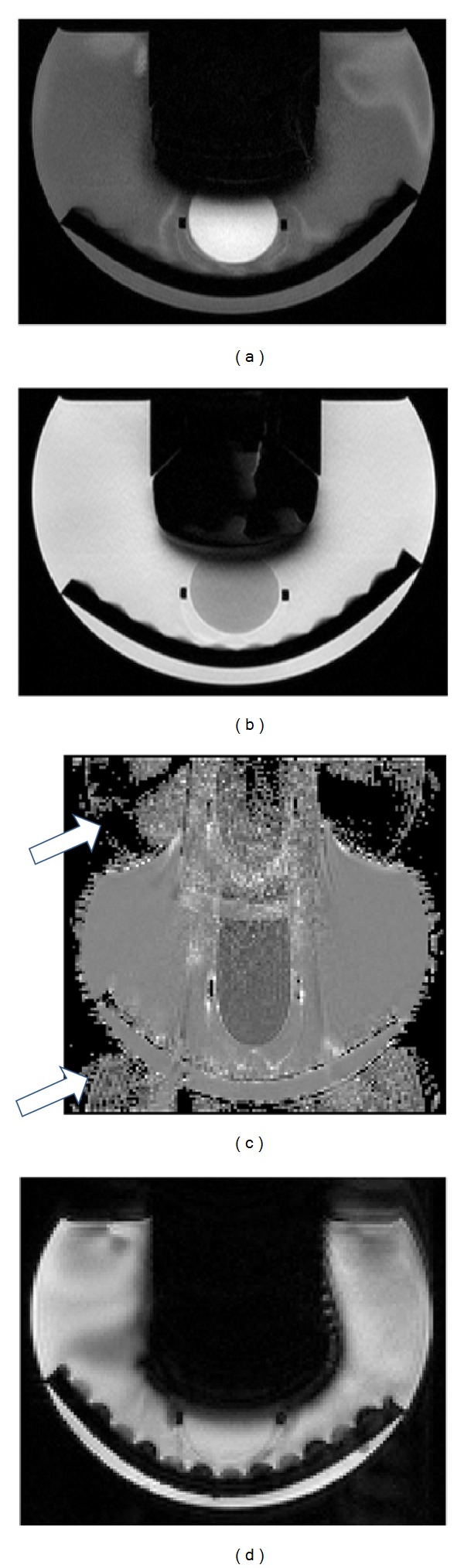
(a) *T*_1_-weighted, (b) *T*_2_-weighted, (c) ADC, and (d) GRE-EPI magnitude images obtained with the US imaging transducer in contact with a cryogel sample in the water tank, with acoustic absorber beneath. The white arrows in (c) indicate ghosting artefacts. The curved structure at the bottom of the images is an acoustic absorber which was placed in the bottom of the water bottle to minimise reflections when acquiring US data. (a), (b), and (d) show a field of view (FOV) measuring 22 × 15 cm and (c) 22 × 20 cm.

**Figure 10:**
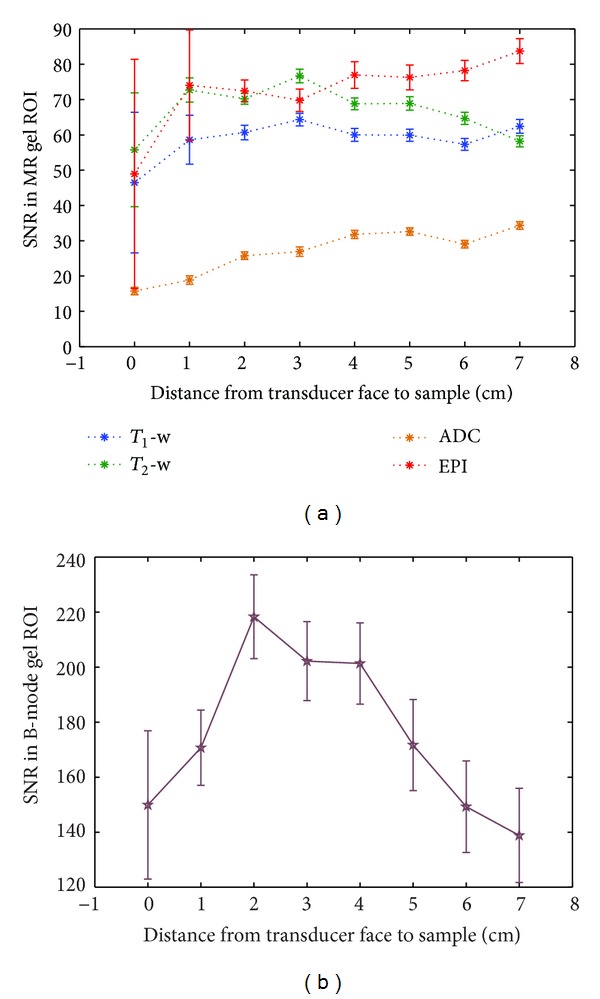
SNR data for (a) MR images from all 4 sequences and (b) B-mode US images, with increasing transducer-sample distance. Error bars represent the percentage uncertainty in intensity within the ROI in a single image frame in each case.

Figure 9 shows *T*_1_- and *T*_2_-weighted image slices, an ADC map, and a segmented EPI image, acquired with the US transducer face, wrapped in a layer of foil, in contact with the top of the cryogel sample. The large black regions in Figures 9(a), 9(b), and 9(d) are due to the magnetic susceptibility of the US transducer, the degree of manifestation depending on the MR sequence used. In the ADC map in Figure 9(c) there is significant geometric distortion, which dominates the image, and ghosting artefacts, indicated by white arrows, are also visible at the top and bottom of the image. Changes in the SNR within the gel with transducer-sample distance are shown in Figure 10(a).

For all sequences, the SNR with the sample at the transducer face is lower than at all other distances (Figure 10(a)), and for *T*_1_-weighted, *T*_2_-weighted, and GRE-EPI magnitude images the uncertainty in SNR at this distance is greater than at any other distance. For the ADC maps and GRE-EPI magnitude images, the SNR is greatest at 7 cm separation, but, for *T*_1_- and *T*_2_-weighted images, the largest value occurs at 3 cm.  Figure 10(b) shows the associated B-mode SNR values, which peak at 2 cm separation and drop off above 4 cm.

**Figure 11:**
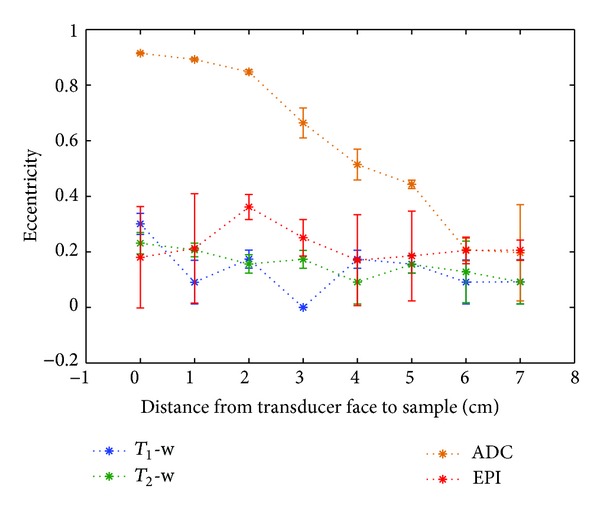
Eccentricity of cross-section of the circular cryogel sample in different MR images obtained as a function of transducer-sample distance. Error bars represent the standard deviation of 3 fits of an ellipse to each image.

The apparent eccentricity of the gel sample due to spatial distortion is plotted as a function of transducer-sample separation in Figure 11. Although there is some variation in the values for all imaging sequences, the ADC maps suffer the most from spatial distortion effects, with the eccentricity approaching 1 at the smallest separations.

### 3.3. Hybrid MR-US Imaging and HIFU* In Vivo*

Doppler US of the femoral artery was attempted. Unfortunately it was not possible to locate the artery using the CH4-1 probe. This was true both in and out of the MR environment. Flow measurements were successfully made using the higher frequency (P10-4) probe, but this was not translatable into the existing hybrid imaging system.

**Figure 12:**
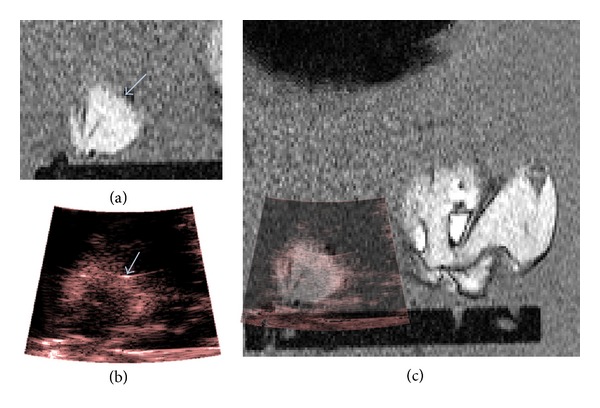
An example 2D *T*_1_-weighted MR image (a) and corresponding B-mode image (b) of the alignment reflector (arrows) used to register MR and US data spatially. The resulting hybrid MR-US image is shown in (c), where the B-mode frame has been set to 50% transparency. The field of view in (c) measures approximately 11 × 11 cm.

MR and US images were taken of the VeroWhite ball target during the* in vivo* alignment process and were manually registered using a translation, as described previously, to create a fused MR-US image. Examples are shown in Figure 12 using a *T*_1_-weighted MR image slice (a) and a B-mode US data set (b). In (c) the animal's abdomen and part of the left leg are visible on the MR image towards the right hand side of the MR image, and the right leg is visible towards the bottom left. The black horizontal bar across the bottom of the MR image is the base of the mounting platform. This corresponds with the highly echogenic horizontal bar close to the bottom of (b).

**Figure 13:**
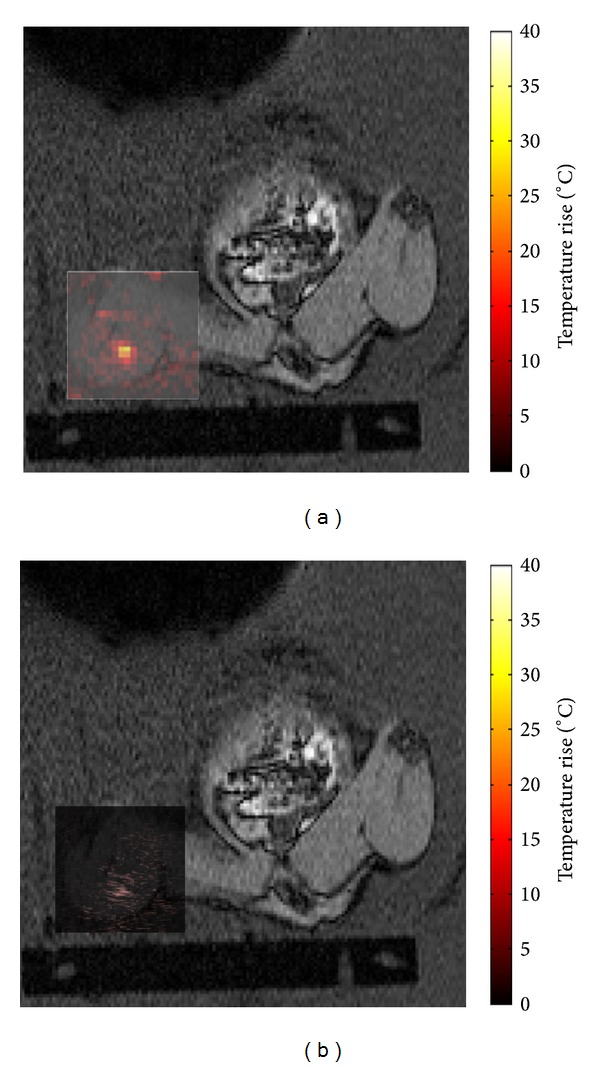
Hybrid *T*_1_-weighted and temperature rise data (a) and *T*_1_-weighted and B-mode subtraction data (b). The field of view shown is approximately 11 × 11 cm.

Figure 13 shows a post-HIFU B-mode image (a) and a temperature map acquired at the time of peak temperature rise (b) over a *T*_1_-weighted image through the focal plane of the HIFU beam. During this exposure, a peak temperature rise of 32 ± 2°C was measured on MR, and an increase in echogenicity was seen at a position corresponding to that of the peak temperature rise.

## 4. Discussion

### 4.1. MR and US Image Noise

RF noise was introduced into the MR scanner by the US imaging transducer when used without any shielding, as demonstrated in Figures 5 and 6. The SNR measured in *T*_1_-weighted images with no US transducer present was not significantly different from that measured with the transducer present and shielded with aluminium foil. In contrast to this, the SNR in *T*_2_-weighted images was reduced by 50% due to the presence of the US transducer, despite the foil shielding. Strong field distortions were introduced by the transducer, causing dephasing, and this effect was found more critical when combined with the TSE *T*_2_-weighted sequence compared with the FLASH GRE *T*_1_-weighted sequence. Caution may therefore be required in certain applications involving *T*_2_-weighted imaging, particularly those where the available SNR within the area of interest is limited. A continuous layer of aluminium foil provided electrical contact with the magnet room door (and hence the MR scanner's RF cage), reducing the amount of RF interference brought into the room by the cable. As a result, the SNR in images of a sample of PVA cryogel was increased by 70–520%, depending on the MR sequence used and the mode of US imaging being used simultaneously (Figure 6). It should be noted that values calculated from the ROI within the gel in the different MR image types were not identical as SNR is highly dependent on the MR sequence parameters. The resulting impact of the foil on B-mode US images was also quantified, and the drop in B-mode image SNR in the gel due to the aluminium foil covering the transducer face was shown to be 24% in the absence of any MR image acquisition (Figure 7). This is likely to be dominated by reflections due to an impedance mismatch at the foil/gel and foil/water interfaces. B-mode SNR values measured in the gel sample as described in the methods section were high when compared with the MR images (an order of magnitude higher), and so the drop in B-mode SNR was not considered to be significantly detrimental for future experiments, given the improvement which the shielding provided in the MR images.

In a B-mode US image there are 2 types of noise: the electronic noise inherent to the system and the speckle pattern which results from interactions of US pulses with densely packed and randomly distributed scatterers in the object under inspection. For an arrangement such as the one used here, where the sample remained stationary with respect to the US transducer, the speckle pattern would remain constant, and only the electronic noise would vary between frames. For this reason the variation in B-mode SNR shown in Figures 6(a) and 6(b) is likely to be due to electronic noise. During an MR scan the switching of magnetic field gradients causes vibrations. As well as inducing acoustic noise, these may also cause motion of the sample within the water tank or of the US transducer mounted on the head coil. During MR scanning using the FLASH and TSE sequences, the variation in B-mode SNR did not exceed that which is seen for an idle MR scanner (Figure 7(b)). This implies that vibrations were not sufficiently high in amplitude to cause changes in the speckle pattern and therefore that scanner vibrations do not influence B-mode imaging in this system.

The SD time trace for the FLASH sequence in Figure 8 shows a quasi-continuous artefact which seems to be made up of a rapid series of bursts of noise, which may originate from audible acoustic noise produced by the MR scanner during the switching of field gradients or from gradient induced vibrations passing from the MR scanner to the sample being imaged. The detected bursts occur every 0.21 seconds. One similar burst is seen in the SD trace taken during the TSE MR sequence, and these were found to occur every 4 seconds. Given TRs of 211 ms and 4 s for the FLASH and TSE sequences, respectively, these bursts coincide with the switching gradients. Additional studies exploring bandwidth and positioning of the transducer within the bore would be required to differentiate between acoustic and vibrational effects, but both are a result of gradient switching. Although the artefacts would interfere with clinical measurements of blood flow, they would not prevent peak flow velocity measurements > 10 cm s^−1^. Further work is required to establish the true clinical impact of the artefacts, which is likely to depend on the vessel under interrogation.

Vertical streaks of noise are visible in the PD images shown in Figure 8. These appear in the water but not in the gel itself, where the SD cursor was placed. It is well known that pulsed diagnostic US beams cause streaming in free fluids [33], and the artefact was also observed outside the MR environment and so can be attributed to motion of the water. PD is inherently more sensitive to motion than CD [34, 35], which explains why the same artefacts are not visible in the CD images in this case.

### 4.2. Transducer-Sample Separation

MR images taken with FLASH, TSE, and GRE-EPI sequences showed that placing the front face of the US imaging transducer in direct contact with a gel sample caused a drop in MR signal close to the transducer, due to the susceptibility artefact it produces (Figure 9). Out of the four sequences tested, segmented GRE-EPI magnitude images showed the greatest drop, as shown in Figure 10(a). *T*_1_-weighted, *T*_2_-weighted, and GRE-EPI magnitude image data also showed large uncertainties in SNR at this position compared with those measured at greater distances. This was also the case at 1 cm separation. Although the ADC maps also had lower SNR for shorter transducer-sample distances, this was likely to be caused by the spreading out of the signal from the gel over a larger area, due to the significant geometric distortion produced by the US transducer and seen in Figure 9(c). Both ADC maps and GRE-EPI magnitude images exhibited the greatest SNR when the transducer-sample separation was at its maximum, 7 cm. The overall trend was an increase in SNR with increasing separation, which implies that the effect of the presence of the US imaging transducer is greatest for these two sequences. Both the FLASH and TSE sequences showed a maximum SNR at 3 cm separation. Beyond this, the FLASH image SNR remained at a roughly constant level, but the TSE image SNR dropped steadily. The reason for this is unclear, and visual inspection of the images used in the calculation did not reveal any potential causes.

SNR data from B-mode images, plotted as a function of transducer-sample separation in Figure 10(b), indicate that the chosen distance is important for US, as well as MR, imaging. The low SNR at zero separation is not surprising as all US transducers have a dead zone close to their surface, in which little or no signal is picked up [36]. The peak B-mode SNR was found to be at 2 cm separation, and beyond 4 cm it reduced consistently. Although the TGC was set prior to all measurements to give the most uniform brightness over the entire image depth, it is possible that differences in the gain at different image depths resulted in differences in the SNR. As the B-mode SNR was an order of magnitude greater than the MR image SNR, because values were always far greater in B-mode images than in any of the MR image types, it was less limiting, particularly as it could easily be altered prior to each study through appropriate adjustment of US imaging parameters.

Geometric distortion effects were seen most clearly in the ADC maps. It is well documented that EPI diffusion-weighted images are prone to distortion due to eddy current induced nonlinearities from the large diffusion-weighted gradients, in addition to static field (*B*_0_) inhomogeneities. Calculation of the eccentricity of the image of the cryogel sample in GRE-EPI magnitude images allowed the distortion to be quantified. A circle has, by definition, an eccentricity of 0. For a transducer-sample separation of 7 cm, all images showed the sample to have an eccentricity of 0.1-0.2, thus minimal distortion. The important result is that reducing the transducer-sample separation caused the sample to appear to stretch towards the transducer in the ADC maps. Eccentricity values for the GRE-EPI magnitude images are higher at 2 and 3 cm separation, but remain close to 0.2 at greater distances, and at 0 and 1 cm separation. It is possible that these images are affected by distortion, but, at 0 and 1 cm, the transducer was close enough to the sample that the large susceptibility artefacts prevented accurate measurement, hiding a significant proportion of the gel outline (Figure 5). *T*_1_- and *T*_2_-weighted images showed greater eccentricities at 0 cm separation than at any other, so it is possible that these may also be susceptible to geometric distortion very close to objects with a high magnetic susceptibility.

Many of the eccentricity measurements had large associated uncertainties. Since the in-plane voxel sizes were larger than for the *T*_1_- and *T*_2_-weighted sequences (1.6 mm for GRE-EPI compared with 0.8 mm for *T*_1_-weighted and 0.4 mm for *T*_2_-weighted), the uncertainty in the fitting of an ellipse to these images of the sample was greater. This was also true for the ADC maps, with an in-plane resolution of 1.6 × 1.6 mm, but the larger sizes of the ellipses caused by the distortion reduced the uncertainty in fitting for the smaller transducer-sample separations. As a result of the findings shown in Figures 10(a) and 11, it was decided that, as a compromise between the practicality of mounting the US imaging transducer and the ability to acquire images with a variety of MR sequences without causing prohibitive loss of signal or geometric distortion, a transducer-sample separation of 4–7 cm should be used.

### 4.3. Hybrid MR-US Imaging and HIFU* In Vivo*

Doppler measurements were not possible using the CH4-1 imaging transducer. Further measurements with a similar transducer with standard cable length would help to identify whether this was due to a lack of sufficient spatial resolution for localisation or due to additional signal attenuation provided by the 8 m cable.

The VeroWhite ball target allowed single-point registration of MR and US images in the *x*-*y* plane. In principle this could be done using anatomical features, but the contrast achieved using the clinical abdominal US imaging probe in the rat thigh was insufficient for this. Figure 12(b) shows the ball target appearing as a horizontal streak in the B-mode image, whilst in the MR image (Figure 12(a)) it is a more circular signal void. This is to be expected, as the US beam reflects strongly from the top of the nylon, and the resolution of US images is typically worse in the lateral direction (perpendicular to the US beam propagation direction) than in the axial direction (along the direction of propagation of the beam). For this reason the registration between MR and US images was more accurate in the vertical direction than the horizontal one. It was estimated that the accuracy of registration was 3 mm horizontally and 1.5 mm vertically, based on the in-plane dimensions of the MR image voxels and the appearance of the VeroWhite target in MR and US images due to the inherent resolution of the imaging techniques. The resolution of the US imaging system was 3 mm laterally and 1 mm axially. This may be improved by the use of higher frequency US imaging, which would consequently also allow the Doppler US measurements in the rat femoral artery. The US scanner manufacturer expressed concerns regarding the ability to transmit signals > 4 MHz along an 8 m length of cable to a system outside the magnet room due to the signal attenuation in the cable degrading the quality of clinical images. Ideally, a portable US imaging system would be used inside the magnet room provided it remained at a safe distance from the magnet and did not introduce further noise into MR images.

This system has demonstrated the ability to monitor both temperature rises and echogenicity changes associated with exposure to HIFU, as shown in Figure 13. The increase in echogenicity was due to bubble activity within the focal region, as verified by passive cavitation detection. These are both important aspects relating to the efficiency and accuracy of HIFU treatments, which cannot both be monitored using currently available clinical HIFU treatment monitoring systems. Work by Viallon et al. has investigated the monitoring of boiling bubbles and temperature rises in phantoms and* ex vivo* tissues, finding that susceptibility related errors can occur in MR temperature rise measurements, but that there is a potential to correct for these using B-mode image data [22]. Furthermore, the information pertaining to bubble locations from US imaging could potentially help to improve the efficiency of treatments by using disruptive boiling to enhance ablation [37]. Further developments to this current system may also allow changes in vessel patency to be detected, making the system ideal for studies of vascular occlusion. Although the current system is limited in preclinical applications due to its low US image resolution, it may prove successful in a clinical setting, where Doppler US data could be acquired in larger blood vessels. The ability to perform MR-US imaging clinically would require comprehensive investigation into aspects of safety, such as the potential to heat the transducer. It would also require the development of more complex apparatus to give improved flexibility in the arrangement of the imaging planes. Better in-depth image registration techniques in 3 orthogonal planes would be required if the 2 modalities were not imaging in precisely the same anatomical orientation. Furthermore, a wide or open bore MR imaging system may also be required in order to provide sufficient space.

## 5. Conclusions

A combined imaging system which allows simultaneous MR and US imaging has been developed using cryogel phantoms and its use has been demonstrated in principle in rats* in vivo*. This was achieved using standard commercially available clinical imaging equipment: a 1.5 T MR scanner and an US imaging system modified with an 8 m cable to ensure conditions were MR safe. Electrical grounding of the transducer to the magnet room's RF shielding was achieved by wrapping it, and its 8 m cable, in a layer of aluminium foil, and partially closing the magnet room door to maintain continuity of the RF shield.

To overcome the susceptibility artefacts produced by the MR-safe US imaging transducer, it was mounted at least 4 cm from the target region of interest. In gel samples there was some evidence of vibration and/or acoustic noise generated by the MR scanner in the US data whilst running various MR imaging sequences, demonstrated by differences in B-mode image SNR and by artefacts in Doppler US data. In SD mode it caused artefacts which could mask Doppler signals.

This study has demonstrated the use of hybrid MR-US imaging for the guidance and monitoring of HIFU exposure beyond motion compensation applications. Further work is required to explore the appearance of tissues on MR-US images before, during, and after HIFU. By combining the soft tissue contrast and accurate thermometry of MR with the real-time capabilities and visualisation of cavitation bubbles and blood flow achievable in B-mode and Doppler ultrasound, such a system shows promise as a useful tool for clinical HIFU guidance and monitoring. Furthermore it may be possible to explore measurements of tissue stiffness changes in response to HIFU. The system would also have many applications in the diagnosis of disease.

## Figures and Tables

**Table 1:** US scanner settings for B-mode and Doppler modes.

US imaging mode	Total image depth (cm)	Centre frequency (MHz)	Pulse repetition freq. (Hz)	Velocity range (cm/s)	Frame rate (Hz)
B-mode	13	4	N/A	N/A	40
Colour/power Doppler (CD/PD)	13	3.3	977	±11	9/11
Spectral Doppler (SD)	N/A	3.3	610	±15	25
